# Mapping immersive digital tools in the ICT4Water Cluster: innovation, interoperability and impact.

**DOI:** 10.12688/openreseurope.21096.2

**Published:** 2025-11-28

**Authors:** Ilias Karachalios, Nikolaos Tantaroudas, Christos Makropoulos

**Affiliations:** 1 Department of Water resources and environmental engineering, School of Civil Engineering, National Technical University of Athens, Athens, Attica, GR 157 80, Greece; 2 Institute of Communication and Computer Systems, Athens, Attica, GR 157 73, Greece

**Keywords:** immersive technologies; virtual reality; augmented reality; mixed reality; extended reality (XR); digital water; interoperability; ICT4Water Cluster

## Abstract

**Background:**

Immersive technologies—virtual, augmented, and mixed reality (XR)—are being piloted in the water sector for public engagement, training, and decision support. Within the European Commission’s ICT4Water Cluster, multiple projects report XR tools, but comparable evidence on functionality, interoperability, and scalability is limited.

**Methods:**

We conducted a two-stage survey across ICT4Water projects (preliminary project-level screening: September–December 2023; tool-level assessment: April–May 2024). Responses were triangulated with public project materials. Seven distinct immersive solutions were analysed using a four-dimension rubric covering functionality, interoperability, scalability, and perceived impact.

**Results:**

The portfolio spans VR, AR, MR, decision-support dashboards, and public-facing installations. Reported deployment options include PCs (4/7; 57%) and smartphones (4/7; 57%), with online-only operation in 3/7 (43%) tools and offline capability in 2/7 (29%). Interoperability is uneven: 3/7 (43%) expose data integration or APIs, while 4/7 (57%) lack external interfaces. Evidence of scalability beyond single-site pilots is reported for 4/7 (57%) tools; 3/7 (43%) remain at pilot/proof-of-concept stage.

**Conclusions:**

XR can enhance awareness, learning, and operational insight, yet broader uptake depends on open interfaces, shared semantics, and post-project maintainability. We recommend designing against open standards and Smart Data Models, adopting modular packaging and open repositories to support post-project sustainment, and embedding XR within utility workflows and digital-twin initiatives to enable responsible scale-up.

## Plain Language Summary

Water systems are complex and mostly hidden - from the pipes under our streets to the treatment plants on the edge of town. This article looks at how immersive digital tools - like virtual reality (VR), augmented reality (AR), and mixed reality (MR) - can make this invisible world easier to see and understand. The research reviewed projects in the European ICT4Water cluster and mapped the tools they created. We found seven distinct tools, ranging from AR apps that help visitors understand water-reuse technologies, to VR training rooms for planners, and MR guides that support first responders during emergencies. These tools turn data and engineering into clear pictures and step-by-step experiences that anyone can follow, not just specialists.

What do they change in practice? People learn faster, discussions between citizens and authorities become more concrete, and operators can spot problems sooner - especially when these tools are connected to live data (so what you see reflects what is happening right now). At the same time, we noticed common hurdles: many apps don’t “talk” to other systems, some need expensive hardware, and several stop being updated after a project ends.

Based on this mapping, we recommend four simple steps: build on open standards so tools connect easily; design for accessibility and multiple languages; plan maintenance from day one so tools outlive short projects; and link immersive visuals to real measurements and digital twins so they are useful in daily decisions.

If we do this, immersive tools can help communities save water, prevent pollution, and run critical services more safely and efficiently.

## Introduction

The digital transformation of the water sector has become a practical driver of smarter, more sustainable resource management. Climate change, rapid urbanisation and rising demand pile fresh pressure onto already stretched water infrastructure, pushing utilities toward data-driven solutions that shorten reaction times and cut losses ^1^. Recent outreach pilots even show citizens using phone-based augmented reality to explore circular-economy options in real-time.

Among the digital advances, immersive tools—virtual, augmented and mixed-reality—are attracting notice for their potential to re-shape water governance, education and day-to-day operations ^2^. Virtual reality (VR) places users inside fully synthetic environments where they can rehearse tasks such as flood-gate sequencing or membrane-cleaning cycles ^3^. Augmented reality (AR) overlays digital information on real-world objects, enhancing field training, public awareness campaigns, and infrastructure maintenance ^4^. An early example of digital engagement at household scale is the NESSIE platform, which provided web-based feedback on water use derived from smart-meter data and analytics ^5^. Similar concepts have since been explored in immersive and AR-enhanced formats building on such data infrastructures, although these developments extend beyond the original NESSIE publications. Mixed reality (MR) more broadly blends digital and physical layers, offering real-time, hands-on decision spaces that can improve collaboration among stakeholders ^6^. In the safety and security domain, the PathoCERT project has demonstrated how multi-modal guidance on smartphones can support first responders during contamination incidents ^7^, and related prototypes within the Cluster are now experimenting with XR-enabled interfaces on top of such platforms. When these visual layers are connected to digital twins - data infrastructures that combine live telemetry, physics-based models and AI surrogates ^8, 9^ - they can unlock predictive analytics and optimisation of complex water networks, even if the underlying publications focus primarily on the data and systems layer rather than on immersive front-ends.

### The role of the ICT4Water cluster

The ICT4Water Cluster, an initiative of the European Commission, brings together more than 60 projects to advance interoperable smart-water solutions ^1^. A core focus is to strengthen stakeholder participation through digital tools that enable actor engagement, scenario-based decision-making and interactive learning ^10– 12^.

Within this framework, the Actor Engagement and Co-Creation Group has undertaken an in-depth assessment of immersive tools created or adopted by Cluster members. In this paper we compile that inventory, and evaluate each tool against four pragmatic criteria, functionality, interoperability, impact and scalability ^2^. Early feedback from developers and end-users highlights key challenges: proprietary data formats, limited accessibility after project close, and the need for structured onboarding and training ^13, 14^. These findings echo cross-sector XR-user studies and point to concrete next steps.

### Objectives of the study

This study aims to:

1. **Identify and catalogue** the immersive digital tools used within the ICT4Water Cluster.
2. **Evaluate their functionality and interoperability** within water management frameworks.
3. **Assess their impact and scalability** across different use cases, including decision support, education, and infrastructure monitoring.
4. **Provide recommendations** to enhance adoption, usability, and effectiveness in addressing water-related challenges.

By achieving these objectives, the study contributes to **digital innovation in water governance**, supporting the EU’s broader policy goals for a sustainable and digitally advanced water sector. The insights gathered will **inform future developments, guide industry stakeholders, and contribute to ongoing research** in immersive digital technologies for water management.

## Literature review

### The digital turn in water management

Digitalisation is steadily reshaping European water services, marrying high-frequency sensing, data analytics and customer apps with decades-old pipes and pumps. The European Commission’s *ICT4Water Action Plan* sketched a “Digital Single Market for Water Services” built on open interfaces, shared vocabularies and cross-utility pilots ^15^. That ambition is echoed in the 2024 *Rolling Plan for ICT Standardisation*, which puts water-management digitalisation on the Green-Deal priority list ^16^.

Yet progress remains uneven, an ethnographic deep dive into Ghana Water Company shows smart meters and mobile billing being bolted on as stand-alone add-ons, limiting value capture and reinforcing organisational silos ^17^. Similar stories surface elsewhere, reminding us that hardware and code do not, by themselves, dismantle institutional inertia.

### Immersive technologies: definitions and capabilities

Immersive media are now on trial across the entire water value chain—from catchment planning to customer engagement. A consolidated summary of definitions, core capabilities, and example use cases is provided in Table 1.

**Table 1.** Immersive modalities, their core affordances, and illustrative use-cases in the water sector.

Modality	Core affordance	Illustrative water sector usecase
**Virtual-reality (VR)**	Full synthetic environment for risk free exploration	Serious game prototype allowing citizens to test flood-mitigation portfolios in a 3-D catchment ^26^
**Augmented-reality** **(AR)**	Data overlays anchored to the physical asset	HoloLens based visualisation of underground sewers to s assist condition assessment ^27^
**Mixed-reality (MR)**	Real time coupling of digital objects and physical space	Field level inspection tool that fuses BIM, IoT and simulation data for water supply pipes ^28^

Beyond operations, these media are valued for communicating complex, dynamic risks to non technical audiences and for shortening training cycles in hazardous settings.

### Digital twins and interoperability in water systems

Digital twins (DTs) augment conventional SCADA setups with live telemetry, physics based simulators and AI surrogates. Recent work demonstrates how a DT of a municipal network can be queried in VR/MR to rehearse incident response or schedule predictive maintenance ^18^. Similar VR walk-throughs are now being trialed on FIWARE-enabled raw-water twins, showing that the concept is not limited to distribution networks ^9^. Early operational evidence is emerging; Gothenburg’s sewer utility, for example, reports pump-energy savings and overflow reductions after wiring its real-time control system to a DT platform ^19^. Whether these gains scale hinges on common semantics. WaterML 2.0 already standardises time-series exchange, yet immersive front ends still struggle to ‘speak’ to heterogeneous back-ends. Wider uptake of linked-data vocabularies such as **SAREF4WATR** and RESTful NGSI-LD APIs would close that gap ^20^.

### Actor engagement, serious gaming, and capacity building

Gameful environments are a hallmark of the EU SIM4NEXUS project, where stakeholders co designed a policy sandbox to explore long term trade offs across the water energy food land climate nexus ^21^. A meta analysis of 41 urban water games confirms their pedagogical value but notes that only one third were assessed with real end users, limiting generalisability ^22^. Beyond the water domain, a recent review of 230 sustainability focused gamification studies highlights the design levers, narrative framing, social competition, real world rewards, that consistently deliver behavioural change ^23^. Importing these insights could strengthen the next generation of digital water engagement tools.

### Gaps in knowledge and need for structured assessment

While pilot studies are everywhere, yet we still lack a panoramic view of how immersive tools perform across regions, governance set-ups and sometimes creaky IT stacks. Comparative metrics for technical readiness, interoperability and socio economic impact are still rare. To fill that gap, we compile a Cluster-wide inventory of immersive applications and, borrowing from the interoperability principles above, we propose a lean rubric others can lift or refine.

## Methodology

This study employed a **mixed-methods approach** combining structured data collection, stakeholder engagement, and comparative analysis to assess immersive digital tools developed or used by members of the ICT4Water Cluster. The methodology was developed under the framework of the *Actor Engagement and Co-Creation Group* and aligns with the ICT4Water Cluster's broader goals of promoting digital transformation through participatory innovation.

### Research design

The assessment was carried out in **two consecutive phases**, involving separate but complementary rounds of data collection:

1. A **Preliminary Questionnaire** ^24^ distributed across the ICT4Water community to gather an initial inventory of immersive digital tools, and
2. A **Main Survey ^25^**, designed to explore specific technical and functional characteristics of each tool, including interoperability, user experience, scalability, and perceived impact.

These instruments were supported by qualitative feedback from respondents, including open-text responses, workshop discussions, and follow-up communications with project representatives. This multi-layered approach provided both breadth and depth in the evaluation.

### Sample and participants

The sampling frame comprised all ICT4Water Cluster projects active or completed at the time of the survey period. Invitations were distributed **via ICT4Water Cluster channels (mailing list)**, with one reminder at a 2-week interval.

- **Preliminary (project-level) questionnaire.** Fielded **September–December 2023**, it targeted **all 77** projects (36 ongoing; 41 past) and yielded **28 valid project responses**, a **36% response rate (28/77)**.
- **Main (tool-level) questionnaire.** Fielded **April–May 2024**, it was addressed to projects that had indicated the use of immersive (XR) tools. It collected **7 valid tool-level responses**, each corresponding to a **distinct immersive solution**.

At the time of data collection, these seven responses represented **all immersive tools self-reported by ICT4Water projects** that met the inclusion criteria, providing a workable snapshot of the ecosystem.

### Survey instruments and indicators

The **Preliminary Survey** captured high-level information, including: (i) existence and name of immersive tool(s); (ii) XR environment type (VR, AR, MR); (iii) application domain (e.g., training, decision-support, education).

The **Main Questionnaire** evaluated each identified tool along four dimensions:

- **Functionality:** degree of immersion, online/offline operation, device compatibility, accessibility features;
- **Interoperability:** interfaces with other software, APIs and data systems, conformance to open standards;
- **Scalability:** readiness beyond pilots, modularity, adaptability to new contexts;
- **Perceived impact:** stakeholder engagement, training effectiveness, decision-making support, end-user feedback.

Items combined Likert-type ratings, multiple-choice selections, and open-ended comments. Metadata captured user categories (e.g., researchers, utilities, citizens), usage frequency, and training requirements. **Full instruments and a codebook are provided as Extended data ^24, 25^**.

### Ethics and consent

In line with institutional guidance at National Technical University of Athens, this minimal-risk professional survey **did not require prior ethics committee approval**, as no special-category personal data were collected, and all responses were anonymized. **Electronic informed consent** was obtained before participants accessed each questionnaire. No IP addresses or geolocation data were stored; free-text responses were screened/redacted prior to data deposit. Preliminary survey contact details are not publicly shared; only aggregated indicators are reported.

### Data analysis

The survey data were analysed through a combination of:

- **Quantitative analysis** (e.g., frequency distributions, cross-tabulations) to identify general trends and tool typologies.
- **Qualitative thematic coding** of open-ended responses to extract emerging themes, user concerns, and best practices.
- **Comparative assessment** of tools across projects to identify patterns in usage, strengths, and limitations.

• Key findings were visualised using summary tables and figures to support cross-case comparison. Tool functionalities and attributes were mapped to a comparative matrix that captures, for each of the seven tools, connectivity and use mode, device compatibility, intended user groups, interoperability, scalability status, perceived impact and reported challenges. This matrix is provided as an extended data file in the associated Zenodo record ^29^, enabling readers to inspect the tool-by-tool overview in full detail.

### Validation and presentation

Findings from the study were presented and validated during the **ICT4Water Annual Cluster Meeting** on **June 19, 2024**, as part of the **Water Innovation Europe** event in Brussels. This provided an opportunity for live feedback, clarification of tool details, and stakeholder input on future priorities. These discussions were incorporated into the final synthesis.

## Results & findings

This section presents the key findings from the two-stage survey process and stakeholder engagement activities conducted across ICT4Water Cluster projects. The results are organized around four main analytical dimensions: (1) inventory and typology of tools, (2) functionality and user experience, (3) interoperability and scalability, and (4) perceived impact and challenges. Insights were drawn from 28 responses to the preliminary questionnaire and 7 detailed tool-level responses from the main questionnaire.

### Inventory and typology of immersive tools

The survey pinpointed **seven** immersive solutions that are either being built or actively used inside ICT4Water projects. For clarity, each entry below pairs the tool with the EU project that created—or now deploys—it, drawing directly on the questionnaire responses presented to the Cluster in June 2024:

- **ARSINOE VR** ( *ARSINOE* project) – a virtual reality environment in which regional planners explore multi hazard climate resilience scenarios and negotiate adaptation pathways.
- **NextGen AR application & authoring tool** ( *NextGen* project) – mobile augmented reality overlays that let Living Lab visitors “see” circular water technologies on top of existing assets; an integrated authoring module allows non coders to publish new AR scenes.
- **iWAYS immersive Decision Support Suite** ( *iWAYS* project) – a web based analytics cockpit whose 3 D dashboards support factory managers in optimising heat and water recovery lines before installation.
- **PathoVIEW AR instruction app** ( *PathoCERT* project) – a phone based AR guide that walks first responders through sampling and disinfection steps during pathogen contamination incidents.
- **NESSIE mixed reality toolset** – delivered in two successive builds:
  ⚬ **NESSIE Platform** ( *iWIDGET* project): a studio based MR prototype for collaborative design of new water infrastructure layouts.
  ⚬ **NESSIE System** ( *B-WaterSmart* project): a field ready evolution that couples a headset with live IoT feeds so on site engineers can inspect hidden assets. Both builds represent iterations of the *same* tool family rather than separate products.
- **Immersive Media eXpriences (IMX) exhibitions** ( *ULTIMATE* project) – a travelling installation that blends VR headsets, large screen projection and live sensor feeds to bring digital water stories to general audiences.

Mapped to their dominant immersive modes, the portfolio distributes evenly across VR, AR, MR, decision support dashboards and public engagement exhibitions, underscoring the Cluster’s strategy of applying extended reality wherever it offers the greatest value—from high level policy planning through factory optimisation to citizen outreach.

### Functionality and user experience

Tool categories were evenly split: VR 2/7 (29%), Digital platform 2/7 (29%), AR 1/7 (14%), MR 1/7 (14%), simulation/DSS 1/7 (14%). The tools vary considerably in terms of immersion levels, user interface design, and technological requirements. In terms of immersion, the two VR environments offer fully synthetic 3D spaces accessed via dedicated headsets or projection rooms, whereas the remaining five tools combine 2D dashboards, AR overlays or mixed-reality views with conventional screens and handheld devices. In practice, this means that ‘immersive’ spans a continuum from high-end room-scale VR to lighter, semi-immersive experiences that run on standard PCs, tablets or smartphones: ^1^

- **Connectivity & Use Mode**:
  ⚬ 3/7 (43%) of tools require internet access.
  ⚬ 2/7 (29%) function offline; 1/7 (14%) support both modes; 1/7 (14%) require on-site installation.
- **Device Compatibility**:
  ⚬ 4/7 (57%) run on PCs;
  ⚬ 3/7 (43%) on tablets;
  ⚬ 4/7 (57%) on smartphones;
  ⚬ 1/7 (14%) require a dedicated VR headset or on-site artefact.
- **Intended User Groups**:
  ⚬ Tools target a wide spectrum of users, including water professionals, researchers, educators, public authorities, and general citizens.

Several respondents highlighted that while immersion enhances engagement, **user-friendliness and onboarding remain key barriers**. Training resources, language accessibility, and hardware availability were mentioned as critical areas for improvement.

### Interoperability and scalability

A key metric in any digital-water roadmap is the ability of tools to **interoperate** with external platforms and scale beyond pilot contexts:

- **Interoperability**:
  ⚬ 3/7 (43%) of tools offer data integration or API-level communication with other systems.
  ⚬ 4/7 (57%) do not currently support interoperability, representing a limitation for wider adoption.
- **Scalability**:
  ⚬ 4/7 (57%) of tools have been tested for scalability, either via multi-site deployment or stress testing.
  ⚬ 3/7 (43%) remain in pilot or proof-of-concept stages, requiring further investment and infrastructure.

These figures suggest that while technical innovation is advancing, **system-level integration remains an obstacle**, particularly for smaller projects or research prototypes.

### Perceived impact and user feedback

Respondents reported several positive outcomes from the use of immersive tools:

- **Enhanced Learning**: Users indicated higher retention and comprehension when using immersive training modules, particularly in VR environments simulating complex systems like wastewater plants or flood risk zones, a finding aligned with an urban-water game meta-analysis ^22^.
- **Increased Engagement**: Tools like serious games and AR storytelling were praised for boosting citizen awareness, particularly in educational campaigns and public consultations.
- **Decision-Making Support**: Several tools enabled multi-scenario visualization and stakeholder co-creation, enhancing the transparency of planning processes.

While these patterns are consistent across multiple respondents, it is important to note that they reflect self-reported perceptions rather than behavioural measurements. The survey did not collect pre/post learning scores, long-term usage statistics or independent usability evaluations, so the impact signals reported here should be interpreted as indicative rather than causal evidence of XR effectiveness.

Despite these strengths, **common challenges** include:

- Limited funding and time to support full deployment,
- Lack of standard training materials and guidance,
- Hardware limitations in field applications (especially for AR).

### Cluster-wide trends and maturity levels

An aggregated view of all responses suggests that immersive technologies in the ICT4Water cluster are in a **transitional phase**. In this analysis we follow the standard nine-level Technology Readiness Level (TRL) scale, where TRL 1–4 correspond to early concepts and proof-of-principle under controlled conditions, TRL 5–7 to prototype development and testing in relevant but still partially controlled environments, and TRL 8–9 to full system demonstration and routine operation in real-world settings.

- Most tools are between **Technology Readiness Levels (TRL) 5–7**, indicating a shift from research to applied development.
- There is a growing interest in **Digital Twins**, often coupled with immersive dashboards or visual simulations (e.g., VR-linked hydraulic models).
- Use cases are moving beyond demonstration toward **multi-stakeholder engagement platforms**, integrating real-time data and AI-assisted analytics.

This comprehensive analysis provides a basis for identifying strategic needs, aligning technological development with stakeholder demand, and advancing immersive tools from innovation to mainstream adoption within digital water governance.

### Limitations and scope of the evidence

Our findings should be interpreted considering several limitations. First, the evidence base is necessarily small: the preliminary project-level survey achieved a 36% response rate (28/77 projects), and only seven tools met the inclusion criteria for the main tool-level questionnaire. As a result, the mapped portfolio should be read as a purposive sample of self-reporting ICT4Water initiatives rather than a statistically representative picture of the entire Cluster or the wider European water sector. Second, all characterisations of functionality, interoperability and maturity are based on self-reported information from project representatives, occasionally triangulated with public deliverables; we did not conduct independent code audits or usability evaluations. Third, the “perceived impact” dimension synthesises qualitative feedback and subjective ratings rather than behavioural or learning outcomes, so claims about the benefits of XR adoption must be understood as indicative, not causal. Finally, several of the authors are affiliated with institutions that developed or deployed some of the tools inventoried through the NESSIE Platform and NESSIE System; while this insider perspective helped interpret technical details, it may also introduce optimism bias. Future work should therefore seek external validation and complementary quantitative studies (e.g. pre/post training assessments, longitudinal uptake metrics) to corroborate and extend these findings.

## Discussion

### Immersive tools added value in the water sector

The findings of this study are showing that immersive tools (VR/AR/MR) help showcase buried assets, subsurface flows, hydraulic behaviour, or the trade-offs behind flood protection. When engineers and operators use **AR overlays** on site, they can visualise pipe alignments, defects and condition states while standing above the network, which speeds up diagnosis and supports safer decisions. Recent work shows how GIS, AR and machine learning can be combined to render sewer condition dynamically, exactly the kind of “seeing is understanding” we aim for 27.

Immersive tools also make **participation** easier. In serious-game and VR settings, people can explore scenarios together (e.g., upstream retention vs. downstream channel works), see consequences, and then discuss trade-offs without needing expert knowledge. A widely cited study reports that a flood-risk serious game improved stakeholder understanding of options in mixed urban–rural catchments, evidence that play, when well-designed, can support informed dialogue ^26^.

At the citizen level, AR can be used as a **lightweight public-engagement** path on phones people already carry. Controlled trials of an AR “eco-game” found gains in sustainability knowledge and pro-environmental attitudes, suggesting that simple, well-tested AR interactions can raise awareness at scale. For water utilities and projects, this matters: AR can be the front door to more complex back-end systems (dashboards, simulations, digital twins) without imposing heavy hardware ^4^.

Finally, immersive front ends are most useful when **connected to live data**. Experience from wastewater utilities shows that when operational **digital twins** feed a visual interface (not always XR, but compatible with XR), operators get better foresight for storms and can reduce overflows or energy use. That is the direction we recommend for water XR: keep the interface simple, but wire it to real measurements and simulations ^19^.

### Technical barriers: interoperability and integration challenges

Most of the tools we inventoried were built as **project pilots**. They work, but many are effectively ‘islands’. From the survey responses, three recurring design patterns emerged behind this lack of interoperability. First, several XR tools were conceived as stand-alone demonstrators, with hard-coded data and no persistent back end or context broker, which makes it difficult to connect them to live telemetry or simulators once the pilot ends. Second, a subset of applications relies on proprietary vendor platforms where external API access is either restricted or out of scope for the project budget, locking immersive front ends into closed ecosystems. Third, time and funding constraints often prevent project teams from refactoring one-off prototypes into modular services with stable, documented APIs. Together, these factors limit reuse and hinder the integration of XR interfaces into digital twins, decision-support systems and wider utility IT stacks. In practice, we see three concrete fixes:

**(a) Use shared context APIs.** The EU’s **NGSI-LD** standard gives a common way to publish and subscribe to dynamic “entities” (assets, sensors, events). ETSI guidance shows how to link external vocabularies to NGSI-LD so different systems can speak the same language. For XR, this means the headset or phone can query a single context endpoint and receive the right attributes (location, status, forecast) without custom adapters for each pilot ^30^.

**(b) Reuse domain vocabularies.** Rather than citing the SAREF portal, we rely on **peer-reviewed SAREF literature**. The SAREF ontology is well documented in a recent Wiley chapter; for the **water domain**, multiple scholarly works apply **SAREF4WATR** to model water assets and observations (e.g., industrial-water knowledge graphs; water-quality and monitoring ontologies; large open knowledge graphs). Using these vocabularies reduces custom mappings and lets XR clients consume consistent semantics across projects ^20, 31– 33^.

**(c) Standardise data models for payloads.** Instead of the FIWARE programme page, we cite **peer-reviewed studies** that operationalise **Smart Data Models** (and FIWARE/NGSI-LD) at scale: city platforms aligned with Smart Data Models, end-to-end FIWARE reference implementations for context-aware analytics, and new Smart Data Models for flood-risk and satellite imagery. These works show how shared payload schemas shorten integration and help XR interfaces stay plug-compatible ^34– 36^.

Finally, our own lineage in web platforms points to a feasible path: earlier **iWIDGET** work already exposed water-use analytics via the web; newer raw-water supply papers define NGSI/NGSI-LD **data models** for key hydraulic entities—exactly the substrate that immersive front-ends can render, provided those endpoints are kept stable beyond project end ^5, 9, 37^.

### Human-centric design and equity of access

Beyond technical integration, **user-friendliness, training support, and accessibility** were frequently cited as gaps by ICT4Water respondents. Immersive tools, while engaging, often assume a level of digital literacy and access to hardware that is not universally available—particularly among smaller utilities or citizen end-users. Previous studies have shown that without deliberate design for inclusivity, digital tools risk reinforcing existing inequalities in environmental governance ^10, 21^.

Moreover, language support, offline modes, and platform neutrality (e.g., web-based vs headset-dependent) are crucial for making immersive experiences broadly usable. These limitations must be addressed through **co-design practices**, involving end users throughout the development cycle ^38^.

### Strategic policy alignment with EU goals

The European Commission has emphasized digital water transformation through key policies including the **Green Deal**, the **Digital Europe Programme**, and the **Digital Water Action Plan**. The ICT4Water Cluster is positioned to play a vital role in operationalizing these goals through scalable, interoperable, and participatory digital tools ^1^.

Immersive technologies can directly contribute to:

- **Meeting Water Framework Directive goals** by facilitating stakeholder participation.
- **Supporting workforce development** through digital training environments.
- **Enhancing adaptive planning** by enabling multi-scenario visualizations in urban water systems.

These benefits underscore the need for funding frameworks that support **maintenance, scaling, and institutional embedding** of immersive tools.

### Recommendations for future development

To fully realize the potential of immersive technologies in water governance, this study proposes the following targeted actions:

1. **Standardise Interoperability**: Support open architectures and shared data formats through alignment with initiatives like the Common European Data Space for Water.
2. **Promote Scalable Architectures**: Encourage modular design, ensuring tools can evolve beyond demonstration environments.
3. **Invest in Inclusive Design**: Prioritize user onboarding, multilingual content, and low-tech accessibility to reduce adoption barriers.
4. **Integrate Tools with Digital Twin Initiatives**: Leverage real-time data environments to deepen the decision-support capacity of immersive tools.
5. **Support Ecosystem Development**: Fund networks of developers, utilities, and researchers to co-create next-generation immersive solutions for water resilience.

## Recommendations

The assessment of immersive digital tools across ICT4Water Cluster projects reveals both substantial opportunities and key barriers. To ensure that these tools evolve from prototypes to impactful digital solutions within water governance, the following targeted recommendations are proposed:

### Foster interoperability through open standards

A significant number of tools assessed lack the ability to integrate with existing digital systems. To address this:

- **Adopt open APIs and standardized protocols**, such as those developed by the Open Geospatial Consortium (OGC), to ensure compatibility with other water platforms and legacy systems.
- Promote compliance with the **Common European Data Space for Water**, encouraging tools to contribute and draw from shared, interoperable datasets ^16^.

### Prioritize scalability and modularity in tool design

Many immersive solutions remain confined to the pilot stage due to architectural limitations. To enable broader deployment:

- Encourage **modular architectures** that allow tools to be adapted and expanded across geographies and stakeholder contexts.
- Provide **funding mechanisms for long-term maintenance and scaling**, including through Horizon Europe and Digital Europe Programmes.

### Improve user experience and accessibility

Barriers to usability—especially for non-experts—can severely limit the adoption of immersive tools. To enhance access:

- Co-design tools with diverse user groups, incorporating feedback from **utilities, educators, and citizens** at early stages ^38^.
- Ensure **accessibility features**, including multilingual support, offline operation, mobile compatibility, and usability for individuals with varying levels of digital literacy.

### Embed immersive tools in digital twin and smart city strategies

To maximize impact and visibility:

- Integrate immersive solutions with **digital twin environments**, enabling real-time monitoring, predictive planning, and collaborative engagement ^8^.
- Align tool development with **smart city initiatives and EU climate adaptation policies**, such as the EU Mission on Climate-Neutral and Smart Cities.

### Support capacity building and institutional uptake

The successful deployment of immersive tools depends on the ecosystem surrounding them:

- Fund **training programs and onboarding materials** for professionals, municipalities, and community organizations.
- Develop a repository of **best practices, case studies, and reusable components**, hosted within the ICT4Water Cluster and disseminated through EU platforms.

### Enable policy integration and long-term planning

To institutionalize immersive technologies:

- Recognize immersive tools as valid methods of **stakeholder engagement under the Water Framework Directive**.
- Encourage national and regional water authorities to include **digital engagement strategies** in their planning processes and procurement guidelines.

By implementing these recommendations, the ICT4Water Cluster and the broader European water community can unlock the full potential of immersive technologies, supporting innovation, inclusivity, and resilience in the face of growing water management challenges.

## Conclusions

This study provides the first cross-cluster assessment of immersive digital tools used within the ICT4Water community, offering new insights into how technologies such as **Virtual Reality (VR)**, **Augmented Reality (AR)**, and **Mixed-reality (MR)** are being applied to support water resilience, stakeholder engagement, and digital transformation.

The findings highlight a growing ecosystem of immersive solutions that span diverse functions, from simulation-based training and scenario planning to interactive public education and participatory co-creation. While the tools demonstrate strong potential for enhancing transparency, inclusivity, and operational awareness, their deployment is often hindered by limited interoperability, low scalability readiness, and uneven user accessibility.

Key barriers such as **fragmented architectures**, **incomplete API integration**, and **a lack of standardised onboarding** point to the need for more coordinated design strategies and alignment with EU-level data and digital policy initiatives. Furthermore, many tools remain in **mid-stage development (TRL 5–7)**, indicating a critical need for funding and technical support to move toward mainstream adoption.

The integration of immersive tools with broader digital water infrastructures, such as **digital twins, IoT networks, and decision support systems**, presents a compelling pathway forward. Equally, embedding these tools in regulatory frameworks and national strategies will be crucial for institutional uptake and long-term sustainability.

Ultimately, this research contributes to the ongoing vision of the ICT4Water Cluster: promoting a digitally empowered, interoperable, and collaborative European water sector. The recommendations provided offer a roadmap for transforming immersive prototypes into impactful, scalable innovations aligned with **European Green Deal objectives** and the **Digital Europe Programme**.

## Ethics and consent

In line with institutional guidance at National Technical University of Athens, this minimal-risk professional survey **did not require prior ethics committee approval**, as no special-category personal data were collected, and all responses were anonymized. **Electronic informed consent** was obtained before participants accessed each questionnaire. No IP addresses or geolocation data were stored; free-text responses were screened/redacted prior to data deposit. Preliminary survey contact details are not publicly shared; only aggregated indicators are reported.

## Data Availability

Project-level responses from the preliminary screening survey (28 projects) are only reported in aggregate form to preserve confidentiality and avoid re-identification. All tool-level variables that underpin the analyses in this article are, however, shared at respondent level in the anonymised main survey dataset, enabling full replication of the results reported here. No previously published third-party datasets were analysed. Zenodo. Dataset: Responses to the Main Survey on Immersive (XR) Tools in ICT4Water Cluster Projects (Anonymised). DOI: https://doi.org/10.5281/zenodo.17073494 ^29^ Respondent-level answers (CSV/XLSX), codebook, README (collection window, anonymisation steps, reuse notes). Data are available under the terms of the Creative Commons Attribution 4.0 International license (CC BY 4.0). Zenodo: Preliminary Survey on Tools Used by ICT4Water Cluster Projects (English). DOI: https://doi.org/10.5281/zenodo.17341824 ^24^ Main Survey on Immersive Tools in ICT4Water Cluster Projects (English). Preliminary survey ICT4Water digital tools research Data are available under the terms of the Creative Commons Attribution 4.0 International license (CC BY 4.0). De-identified data are shared; no IP addresses or geolocation were stored. Free-text fields were screened/redacted to prevent re-identification. (See Ethics & consent.) No new software was developed.

## Footnotes

^1^Percentages refer to seven detailed responses; multiple selections were possible.
