# Supplementary material for: Mapping immersive digital tools in the ICT4Water Cluster: innovation, interoperability and impact
Source: Open Res Eur. 2025 Nov 28;5:329. Originally published 2025 Oct 28. [Version 2] doi: 10.12688/openreseurope.21096.2 (PMC12699209; doi:10.12688/openreseurope.21096.2)
Supplement: Peer review history

# Reviewer report: Reviewer response for version 2

**DOI:** 10.21956/openreseurope.23865.r65002

**Published:** 2025-12-11

**Authors:** Yutaro Onuki

**Affiliations:** 1 Seijo University, Tokyo, Japan

I have carefully read the revised manuscript and the authors’ responses. All of my previous major and minor concerns have been adequately addressed.

Is the study design appropriate and does the work have academic merit?

Yes

Is the work clearly and accurately presented and does it cite the current literature?

Partly

If applicable, is the statistical analysis and its interpretation appropriate?

Not applicable

Are all the source data underlying the results available to ensure full reproducibility?

Yes

Are the conclusions drawn adequately supported by the results?

Partly

Are sufficient details of methods and analysis provided to allow replication by others?

Yes

Reviewer Expertise:

Cognitive Science, Judgement and Decision-making, and Human-Computer Interaction.

I confirm that I have read this submission and believe that I have an appropriate level of expertise to confirm that it is of an acceptable scientific standard.

# Reviewer report: Reviewer response for version 1

**DOI:** 10.21956/openreseurope.22819.r63690

**Published:** 2025-11-25

**Authors:** Yutaro Onuki

**Affiliations:** 1 Seijo University, Tokyo, Japan

This manuscript provides a valuable contribution by systematically mapping immersive tools (VR/AR/MR) developed and used within the ICT4Water Cluster, and by organising their functionality, interoperability, scalability, and impact along four analytical dimensions. The two-stage survey design, combined with feedback gathered at the annual Cluster meeting, allows the authors to show that immersive tools bring clear benefits, but also to surface important challenges such as “not connecting to other systems,” “remaining at pilot/prototype stage,” and “not being accessible to all user groups.” The recommendations proposed in the manuscript have the potential to serve as practical guidance for future work within the Cluster, especially for addressing these issues.

 At the same time, I believe that the manuscript’s academic rigour and persuasive power could be further strengthened by addressing the points below. I group my comments into Major Points and Minor Points.

 Major Points

 1.

 As described in the Methodology section:

 The Preliminary Survey achieved a 36% response rate (28 out of 77 projects), and the Main Survey collected detailed tool-level responses for 7 tools.

 The authors themselves characterise the results as “a workable snapshot.” At the same time, this sample size is necessarily limited for drawing conclusions that might be interpreted as representative of the ICT4Water Cluster as a whole, let alone the wider European water sector.

 To help readers correctly interpret the scope of the findings, I recommend that the Discussion section explicitly states at least the limitations and potential biases arising from the small sample size. This kind of explicit limitation statement would, in my view, strengthen rather than weaken the credibility of the manuscript.

 2.

 In the Introduction, several concrete examples (NESSIE, PathoVIEW, FIWARE) are presented, but their descriptions do not fully align with the content of the cited references. I recommend checking and, if necessary, adjusting the following:

- NESSIE
  The manuscript cites NESSIE as an AR example:
*“An early example is the NESSIE household-efficiency platform, which coupled AR overlays with smart-meter feedback [5].”*
  However, in the cited paper [5] (Makropoulos et al., 2014), I could not find any explicit discussion of AR overlays.
- PathoVIEW
  The manuscript presents PathoVIEW as an MR example:
*“The forthcoming PathoVIEW suite for first responders illustrates how MR can also support crisis management [7].”*
  Yet the cited work [7] (Knecht & Kosuru, 2024) does not read as a study centred on mixed-reality headsets or spatial MR environments.
- FIWARE
  In the “Digital twins and interoperability in water systems” section, FIWARE is used as a VR example:
*“Similar VR walk-throughs are now being trialed on FIWARE-enabled raw-water twins, showing that the concept is not limited to distribution networks [9].”*
  However, the cited paper [9] (Kossieris et al., 2021) does not provide details on VR walk-through implementations or evaluations.

My interpretation is that these references are mainly intended to demonstrate the existence of platforms or data models, while the AR/MR/VR elements may be based on subsequent developments or related work. In their current form, however, the sentences can easily be read as if the cited papers themselves directly report AR/MR/VR implementations.

 I therefore recommend either:

- providing more appropriate references that explicitly describe the AR/MR/VR implementations in question, or
- splitting the sentences so that one part clearly cites the platform or data model (NESSIE, PathoVIEW, FIWARE), and a separate part discusses AR/MR/VR usage or potential, without implying that the same reference directly covers both.

This would make the relationship between examples and references more transparent and prevent misunderstandings for readers who follow up the citations.

 Minor Points

 1.

 In the Results section, the characteristics of the seven tools are carefully described in text. However, this information is currently dispersed across multiple paragraphs, which makes side-by-side comparison somewhat demanding for the reader.

 I recommend adding a summary table in which:

- Rows: the seven tools (e.g. ARSINOE VR, NextGen AR application, iWAYS suite, PathoVIEW, NESSIE toolset, IMX exhibitions)
- Columns, for example:

• Connectivity & use mode

 • Device compatibility

 • Intended user groups

 • Interoperability

 • Scalability status

 • Perceived impact and user feedback

 • Technology Readiness Level

 • Challenges

 Such a table, complementing the qualitative text, would make similarities and differences among tools more immediately visible and would reinforce one of the core contributions of the paper: the cross-cluster overview.

 If space or layout constraints make it difficult to include such a table in the main text, an alternative would be to refer explicitly in the Results section to the comparative matrix already provided in the Zenodo dataset (e.g. the Excel sheet of “Immersive tools of ICT4Water Cluster (Answers).xlsx”). A brief pointer there would help readers quickly locate the tool-by-tool overview, even if it remains in the extended data rather than in the article body.

 2.

 In the “Cluster-wide trends and maturity levels” subsection, the statement that:

 “Most tools are between Technology Readiness Levels (TRL) 5–7 …”

 is one of the important findings of the paper. However, some readers may not know what TRL 5–7 implies in practice.

 I suggest including a brief explanation of the TRL scale at one point in the manuscript, for example:

- TRL 1–4: from early concept to proof-of-principle under controlled conditions.
- TRL 5–7: prototype development and testing under relevant but still controlled conditions.
- TRL 8–9: system demonstration and operation in real or operational environments.

Then you can state that most tools in this study fall into TRL 5–7, meaning that prototypes exist and are being tested in relevant or near-operational contexts. This would help non-expert readers appreciate the significance of the maturity assessment.

 3.

 In the “Functionality and user experience” section, the manuscript notes that:

 “The tools vary considerably in terms of immersion levels …”

 but immediately afterwards there is no direct quantitative or categorical breakdown of immersion levels. Since immersion is one of the key dimensions distinguishing VR, AR, and MR, a small amount of additional detail here would improve clarity. Even one or two sentences summarising this breakdown would make it easier for readers to understand in what sense “immersion levels” differ across the portfolio.]

 Thank you very much for your assistance.

Is the study design appropriate and does the work have academic merit?

Yes

Is the work clearly and accurately presented and does it cite the current literature?

Partly

If applicable, is the statistical analysis and its interpretation appropriate?

Not applicable

Are all the source data underlying the results available to ensure full reproducibility?

Yes

Are the conclusions drawn adequately supported by the results?

Partly

Are sufficient details of methods and analysis provided to allow replication by others?

Yes

Reviewer Expertise:

Cognitive Science, Judgement and Decision-making, and Human-Computer Interaction.

I confirm that I have read this submission and believe that I have an appropriate level of expertise to confirm that it is of an acceptable scientific standard, however I have significant reservations, as outlined above.

# Response

**Published:** 2025-11-25

**Authors:** ilias karachalios

**Major Point 1 – Scope and limitations of the sample**

 As noted in our response to Reviewer 1, we have added a dedicated subsection in the Discussion titled “Limitations and scope of the evidence” where we: highlight the response rate of the preliminary survey and the small number of tools in the main survey; clarify that the paper presents a workable snapshot of self-reporting projects rather than a representative sample of the entire ICT4Water Cluster; explain that self-selection and the self-reported nature of the data may bias the picture towards projects with stronger communication resources. We believe this addition makes the boundaries of our evidence base more transparent.

**Major Point 2 – Alignment between examples (NESSIE, PathoVIEW, FIWARE) and cited references**

 Our intention was to use these citations to anchor the underlying platforms and data infrastructures, while mentioning immersive or mixed-reality developments that build on them. To avoid any confusion: We have rewritten the relevant paragraph in the Introduction. NESSIE is now presented primarily as a platform providing web-based feedback on water use derived from smart-meter data, with a brief remark that immersive and AR-enhanced formats have later been explored as extensions of such infrastructures, without implying that these are part of the original publication. PathoCERT / PathoVIEW are described as providing multi-modal guidance for first responders via smartphones, with XR interfaces mentioned as ongoing or related experimentation rather than as the core contribution of the cited work. FIWARE and NGSI-LD are explicitly framed as data and context-broker infrastructures which can support VR or 3D dashboards, again without suggesting that the referenced papers themselves include VR evaluations. We have kept all the original references, but we have now separated more clearly which part of the sentence is covered by the citation and which part refers to subsequent or potential XR usage. We hope this resolves the ambiguity identified by the reviewer.

**Minor Point 1 – Summary table / comparative matrix**

 In the revised “Data analysis” subsection we now: explicitly describe the comparative matrix used to aggregate tool-level attributes (connectivity and use mode, device compatibility, intended user groups, interoperability, scalability status, perceived impact and challenges); refer directly to the inputs at the associated Zenodo record, noting that this matrix enables readers to inspect the tool-by-tool overview in full detail. If the editorial team considers it useful, we would also be happy to format a condensed version of this matrix as an additional table in the main text in a further iteration, but we believe the strengthened link to the extended data already addresses the reviewer’s request.

**Minor Point 2 – Explanation of the TRL scale**

 In the revised Results subsection “Cluster-wide trends and maturity levels”, we now include a concise explanation of the nine-level TRL scale immediately before discussing our findings. Specifically, we note that: TRL 1–4 correspond to early concepts and proof-of-principle under controlled conditions; TRL 5–7 correspond to prototype development and testing in relevant but still partially controlled environments; TRL 8–9 correspond to full system demonstration and routine operation. We then reiterate that most tools in our sample are at TRL 5–7, meaning that they have functioning prototypes being tested in relevant or near-operational contexts rather than being fully embedded in routine utility workflows. We hope this makes the maturity assessment clearer, especially for non-specialist readers.

**Minor Point 3 – Clarifying “immersion levels”**

 In the “Functionality and user experience” section we have expanded the text to clarify the distribution of immersion levels: we specify that two tools in our sample provide fully immersive VR environments accessed through dedicated headsets or projection rooms; the remaining five tools combine 2D dashboards, AR overlays or mixed-reality views with standard screens and handheld devices; we also state that the portfolio spans a continuum from high-end room-scale VR to lighter, semi-immersive experiences that run on standard PCs, tablets or smartphones. This additional detail should make the variation in immersion more tangible. Additional clarifications introduced in response to both reviewers Although not directly requested in single comments, we introduced two small clarifications that we believe improve transparency: In “Perceived impact and user feedback” we explicitly state that impact findings are based on self-reported perceptions, and that no pre/post tests or behavioural metrics were collected. In “Data and software availability” we clarify that preliminary project-level survey responses are only reported in aggregate form to protect confidentiality, while all tool-level variables used in the analyses are shared at respondent level in the anonymised main survey dataset, allowing full replication. We hope that these revisions address all the issues raised by both the reviewers and that the manuscript is now suitable for approval.

# Reviewer report: Reviewer response for version 1

**DOI:** 10.21956/openreseurope.22819.r63185

**Published:** 2025-11-20

**Authors:** Francesco De Paola

**Affiliations:** 1 University of Naples Federico II, Napoli, Italy

Although the seven tools analyzed are representative of current ICT4Water projects, the sample remains small relative to the cluster’s 60+ initiatives. The authors acknowledge that the dataset reflects only self-reported tools, potentially excluding under-documented or non-respondent projects. This limits generalizability and may skew toward projects with greater communication capacity or resources.

 While qualitative feedback indicates improved learning, engagement, and decision-making, no empirical performance metrics (e.g., pre/post-training scores, behavioural data) are reported. The “perceived impact” dimension relies on self-assessment rather than objective evaluation. This weakens claims about the measurable benefits of XR adoption.

 The study identifies interoperability gaps—only 43% of tools expose APIs—but does not deeply analyze technical causes or architectural designs behind these limitations. A comparative case analysis or technical deep-dive (e.g., data model architectures, protocol mapping) would enhance understanding of integration barriers.

 The recommendations—such as promoting open standards or inclusive design—are valid but somewhat generic. The paper would benefit from specific implementation pathways, such as referencing concrete interoperability frameworks (e.g., NGSI-LD data models, OGC APIs) in use by particular tools.

 The authors—who are also involved in ICT4Water projects—acknowledge this but do not reflect deeply on possible bias in tool assessment. Independent validation or external peer benchmarking would strengthen credibility.

Is the study design appropriate and does the work have academic merit?

Yes

Is the work clearly and accurately presented and does it cite the current literature?

Partly

If applicable, is the statistical analysis and its interpretation appropriate?

Yes

Are all the source data underlying the results available to ensure full reproducibility?

Partly

Are the conclusions drawn adequately supported by the results?

Partly

Are sufficient details of methods and analysis provided to allow replication by others?

Partly

Reviewer Expertise:

Hydraulic Engineering

I confirm that I have read this submission and believe that I have an appropriate level of expertise to confirm that it is of an acceptable scientific standard.

# Response

**Published:** 2025-11-25

**Authors:** ilias karachalios

1.  Sample size, representativeness and self-selection

 We have now made these limitations explicit in the manuscript. In the revised version, we have added a dedicated subsection in the Discussion titled “Limitations and scope of the evidence”. There we clarify: that the preliminary survey achieved a 36% response rate (28/77 projects) and that only seven tools met the inclusion criteria for the main tool-level survey; that the resulting portfolio should be read as a purposive sample of self-reporting ICT4Water initiatives, not as a statistically representative picture of the entire Cluster or the wider European water sector; that all characterisations of functionality, interoperability and maturity are based on self-reported information from project representatives, occasionally triangulated with public deliverables. We believe this addition helps readers interpret the findings with the appropriate level of caution.  

 2. Lack of empirical performance metrics and reliance on perceived impact

 Our aim was to surface how project teams and users perceive the contribution of immersive tools rather than to provide causal evidence of impact. To make this clear:

1. In the Results subsection *“*Perceived impact and user feedback *”* we have inserted a short paragraph explicitly stating that:
  - the impact patterns reported are self-reported perceptions;
  - no pre/post learning scores, longitudinal usage statistics, or independent usability evaluations were collected;
  - the impact signals should therefore be interpreted as indicative rather than causal evidence.
2. In the new “Limitations and scope of the evidence” subsection we reiterate this point at manuscript level, noting that future work should include quantitative assessments (e.g. pre/post training assessments, longitudinal uptake metrics) to corroborate our findings.

3. Limited analysis of the technical causes of interoperability gaps

 We have now expanded the subsection “Technical barriers: interoperability and integration challenges” in the Discussion. In the revised text we describe three recurring design patterns that emerged from the survey responses:

1. Stand-alone demonstrators: several XR tools were built as stand-alone visual demonstrators, with hard-coded data and no persistent backend or context broker, which limits connection to live telemetry or simulators after the project ends.
2. Dependence on proprietary platforms: a subset of tools relies on vendor platforms where external API access is restricted or out of scope for the project budget, effectively locking immersive front-ends into closed ecosystems.
3. Resource constraints for refactoring: time and funding constraints often prevent refactoring project prototypes into modular services with stable, documented APIs.

We then connect these observed patterns with the remedies that were already discussed (use of shared context brokers, NGSI-LD / SAREF / Smart Data Models, and open APIs). This should provide a clearer explanation of *why* interoperability is challenging, not only *how* it could be improved.  

 4. Specificity of recommendations and reference to concrete interoperability frameworks

 In the original version we already referred to NGSI-LD, FIWARE context brokers and Smart Data Models, but we agree that the link between these frameworks and our recommendations could be made more explicit. In the revised Discussion:

- we make clearer that the tools which reported higher interoperability typically build on web-based dashboards or decision-support suites connected to IoT platforms and digital twins;
- we explicitly mention NGSI-LD-compliant context brokers, OGC-inspired APIs and domain-specific data models (such as SAREF and Smart Data Models for Water) as concrete examples of infrastructures that can support reusable XR front-ends.

This provides a more tangible implementation pathway, in line with the reviewer’s suggestion.  

 5. Potential bias arising from the authors’ involvement in ICT4Water projects

 In the new “Limitations and scope of the evidence” subsection we add that:

- several of the authors are affiliated with institutions that developed or deployed some of the tools inventoried through the NESSIE Platform and NESSIE System;
- this insider perspective helped us interpret technical details but may introduce optimism bias;
- future work should therefore seek external validation and peer benchmarking to strengthen credibility.

We hope this addresses your concern.
